# The Impact of Foaming Effect on the Physical and Mechanical Properties of Foam Glasses with Molecular-Level Insights

**DOI:** 10.3390/molecules27030876

**Published:** 2022-01-27

**Authors:** Chenxi Zhai, Yang Yu, Yumei Zhu, Jing Zhang, Ying Zhong, Jingjie Yeo, Mingchao Wang

**Affiliations:** 1 Key Laboratory for Advanced Ceramics and Machining Technology of Ministry of Education, School of Materials Science and Engineering, Tianjin University, Tianjin 300072, China; zhangshishi199@126.com (J.Z.); lijianqiao145@163.com (Y.Z.); 2 Sibley School of Mechanical and Aerospace Engineering, Cornell University, Ithaca, NY 14853, USA; jingjieyeo@cornell.edu; 3 Centre for Infrastructure Engineering, Western Sydney University, Penrith, NSW 2751, Australia; 4 College of Science, Civil Aviation University of China, Tianjin 300300, China; mingchaowang0@163.com

**Keywords:** foam glass, mechanical property, foaming effect, molecular dynamics, pore structure

## Abstract

Foaming effect strongly impacts the physical and mechanical properties of foam glass materials, but an understanding of its mechanism especially at the molecular level is still limited. In this study, the foaming effects of dextrin, a mixture of dextrin and carbon, and different carbon allotropes are investigated with respect to surface morphology as well as physical and mechanical properties, in which 1 wt.% carbon black is identified as an optimal choice for a well-balanced material property. More importantly, the different foaming effects are elucidated by all-atomistic molecular dynamics simulations with molecular-level insights into the structure–property relationships. The results show that smaller pores and more uniform pore structure benefit the mechanical properties of the foam glass samples. The foam glass samples show excellent chemical and thermal stability with 1 wt.% carbon as the foaming agent. Furthermore, the foaming effects of CaSO_4_ and Na_2_HPO_4_ are investigated, which both create more uniform pore structures. This work may inspire more systematic approaches to control foaming effect for customized engineering needs by establishing molecular-level structure–property–process relationships, thereby, leading to efficient production of foam glass materials with desired foaming effects.

## 1. Introduction

Foam glass production has emerged to be an effective and efficient approach to recycling mass-produced industrial waste. As compared with conventional condensed hard materials [1,2], the properties of foam glass material include an excellent combination of high mechanical strength [3], low bulk density [4], as well as chemical [4] and thermal [5] stability by virtue of a number of pores inside the foam glass material, which lead to significant applications in building materials [5,6], thermal storage [5], and corrosion resistance [3]. Sheet glass cullet [7], waste glass [8], cathode-ray-tube panel [9], and coal fly ash [7,10] can be recycled to manufacture foam glass materials. The effects of a number of chemical additions, such as chromic oxide, cobaltous oxide, antimonous oxide, manganese dioxide, lead oxide, and antimony trioxide, have been studied to improve the physical and mechanical properties of foam glass materials [6,11,12,13,14,15]. However, efficient preparation of foam glass materials with high performance and low cost, such as high strength and low density with a low sintering temperature and commercially inexpensive foaming agents and additions, remains to be a challenge. This is primarily due to a lack of understanding of the structure–property–process relationships of the foaming effect at the molecular level. Although recycling waste glass can turn waste into something useful, it is challenging to determine the initial mixture composition in glass wastes, which complicates the understanding of the foaming effect and structure-property relationships. Therefore, it is still necessary to prepare foam glass materials with pure raw chemicals to attain clear structure–property–process relationships at the molecular level, thereby, leading to approaches to preparing foam glass materials with superior properties and lower cost. A high sintering temperature of 1500 ℃ is usually needed [16] to yield superior mechanical performance. We previously prepared types of foam glass samples with selected agents and additions [15,17] with similar or even superior physical properties and mechanical performance with much lower sintering temperatures of only 775 ℃ or 780 ℃, which were cost-effective and environmentally friendly. However, there is a limited fundamental understanding of the nature of the foaming effect at the molecular level, which delays an efficient experimental design of high-performance foam glass materials by effectively screening foaming agents and additions at a low sintering temperature.

To address the above challenges, in this study, first, we studied the foaming effect of dextrin, a mixture of dextrin and carbon, and different carbon allotropes by characterizing surface morphology with scanning electron microscopy (SEM) and physical and mechanical properties, in which 1 wt.% carbon black was identified as an optimal choice for a balanced material property. More importantly, the different foaming effects were elucidated at the molecular level with all-atomistic molecular dynamics (AAMD) simulations, which led to molecular-level insights into the structure–property relationships. Radial distribution function (RDF) and number density profile (NDP) were computed; smaller pores and a more uniform pore structure were found to benefit the mechanical behaviors of the foam glass samples. The foam glass samples showed excellent chemical and thermal stability with carbon as the foaming agent. Finally, the foaming effect of foaming promoter CaSO_4_ and foaming stabilizer Na_2_HPO_4_ were explored, and both led to a more uniform pore structure. By providing insight into the molecular mechanism of foaming effect, this work is expected to inspire more systematic approaches to control foaming effect for diversified engineering needs by establishing structure–property–process relationships at the molecular level, thereby, leading to efficient production of high-performance foam glass materials in a bottom-up manner without the conventional trial-and-error approaches.

## 2. Results

### 2.1. Foaming Effects of Dextrin and Different Carbon Allotropes with Molecular-Level Insights

On the basis of our previous studies [17], the effects of foaming agents on the physical and mechanical properties of foam glass materials is significant. Dextrin (${\left[C_{6}H_{10}O_{5}\right]}_{n}$) is one kind of oxidation-type foaming agent that decomposes at high temperatures and releases gas to create pore structures. Oxidation-type foaming agents usually facilitate closed-pore structures in glass melts, leading to lower density which is suitable for artificial floating material needs [4]. First, we calculated the amounts of foaming agent needed to obtain a superior sample density of 0.35 g/cm^3^, as per our previous work [17], due to the empirical carbon loss of ~60% during the sintering. This led to the amount of carbon 1 wt.% [17], dextrin 2.5 wt.%, or a mixture of carbon 0.5 wt.% and dextrin 1.75 wt.%, together with the previously determined basic composition of H_3_BO_3_ 13 wt.%, SiO_2_ 60 wt.%, Na_2_CO_3_ 17 wt.%, K_2_CO_3_ 5 wt.%, and Al_2_O_3_ 5 wt.% for the foam glass samples [17]. Then, we systematically investigated the compressive strength, density, porosity, and volume-absorption rate of the prepared foam glass samples (Table 1). The samples with pure dextrin as the foaming agent showed relatively low strength and density as compared with those using pure carbon as the foaming agent [17]. Nonetheless, dextrin can enable a relatively lower volume-absorption rate (1.15%) which is beneficial for applications based on water. Thus, the dextrin-agent foam glass samples may be more chemically stabilized, but are not a superior candidate for products that require high strength. Interestingly, the mixture foaming agent could provide intermediate physical and mechanical properties, which are suitable for applications that require a balanced material property.

**Figure 1:**
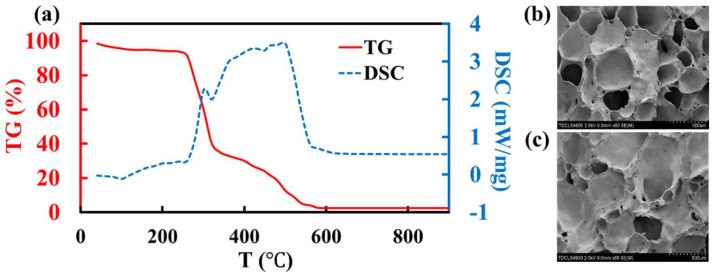
(**a**) DSC–TG of dextrin showing a proper foaming temperature range of 300–550 °C. Pore surface morphology of the foam glass samples by SEM with (**b**) dextrin and (**c**) a mixture of dextrin and carbon as foaming agent under a sintering temperature of 780 °C.

Thermogravimetric and differential scanning calorimetry (TG-DSC) were used to measure the thermal behaviors of the dextrin foaming agent to analyze the cause of the different material properties (Figure 1a). The thermal decomposition of dextrin starts from 300 °C, where there is a small heat-release peak accompanied by a sharp decrease in weight. Then, the oxidation is complete at 550 °C, where the TG curve converges to 0. This indicates that the foaming temperature of dextrin is roughly at 300–550 °C. Compared with carbon [17], the foaming temperature of dextrin is lower, which does not correspond well with the melting and softening temperature (700 ℃) of the foam glass raw mixtures without foaming agents. Foaming that occurs too early leads to significant gas loss without being consumed by the raw mixture, thereby, resulting in inferior performances. We also characterized the surface morphology of the foam glass samples with SEM to investigate the pore structures (Figure 1b,c) under a sintering temperature of 780 °C. Most of the pore diameters are 0.25–0.50 mm with an intermediate pore wall thickness. However, there are more interconnected pores in the samples when dextrin is the foaming agent (Figure 1b), leading to inferior strength and corrosion resistance. In addition, the pore structure is not uniform with the mixture as the foaming agent (Figure 1c), thereby, not harnessing the advantages of porous materials. This may be due to the fact that the wettability between dextrin and carbon is weak, which leads to low diffusivity at high temperatures during sintering, thereby, resulting in a nonuniform foaming.

**Figure 2:**
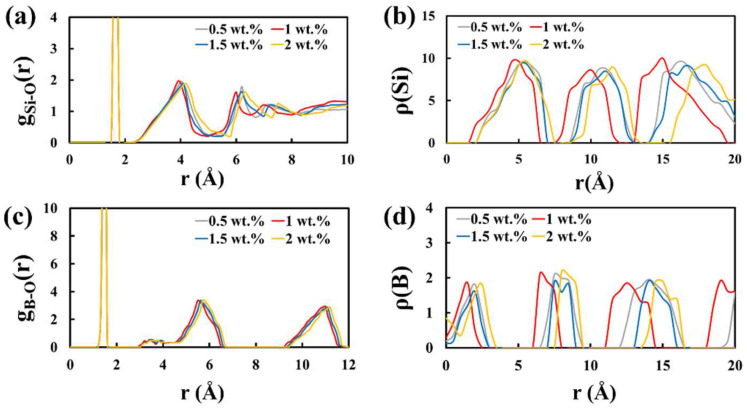
RDFs of (**a**) Si-O and (**c**) B-O, and NDPs of (**b**) Si and (**d**) B, at a representative direction of [1 1 1], of the foam glass samples with different amounts of carbon (0.5/1/1.5/2 wt.%) as foaming agent, under a sintering temperature of 780 °C.

MD simulations are effective tools [18,19,20,21] for probing the material structures and behaviors in an especially small spatiotemporal scale that is beyond the reach of conventional experimental facilities. Considering that carbon as a foaming agent is still superior to dextrin and the mixture of carbon and dextrin, in terms of product strength [17], we performed MD simulations (details in Materials and Methods) to elucidate our chosen optimal amount of carbon (i.e., 1 wt.%) [17] with molecular-level insights into the mechanism. RDFs and NDPs were calculated to characterize the molecular structural configuration of the carbon-agent foam glass samples after sufficient equilibration of the system (see computational procedures in Materials and Methods). Here, g_Si-O_/g_B-O_ is used to denote the RDF of Si-O/B-O pair, and ρ(Si)/ρ(B) is used to denote the NDP of Si/B with different amounts of carbon (0.5, 1, 1.5, and 2 wt.%) added. The first major peak at around 1.6 Å (Figure 2a) shows typical Si-O tetrahedra in the glass structure. After that, the major peak locations move to the left with an increase in carbon amount (from 0.5 wt.% to 1 wt.%), and then move to the right with an increase in carbon amount (from 1 wt.% to 2 wt.%). This may indicate that when 1 wt.% carbon is added, the distances among different Si-O tetrahedra are the shortest as compared with other carbon amounts, implying smaller gas pores inside the sample. Smaller pores could lead to more uniform gas pore structure. NDPs at a representative direction of [1 1 1] for Si atoms are calculated (Figure 2b), which also show the shortest peak location distances (~5 Å) for the 1 wt.% carbon-agent samples as compared with the other carbon amounts. This NDP of Si atoms corresponds well with the RDF of Si-O pairs regarding different amounts of carbon added, which verifies the RDF findings. Furthermore, we calculated the RDFs of B-O pairs and NDPs of B atoms with respect to different carbon amounts (Figure 2c,d). For the RDF (Figure 2c), the major peak at around 1.5 Å shows typical B-O tetrahedra. After that, similarly, distances among major peak locations of 1 wt.% carbon-agent samples are smallest as compared with other carbon amounts, which is further verified by the NDPs of B atoms (Figure 2d). This suggests that more larger pores are formed in the 0.5/1.5/2 wt.% carbon-agent glass samples, occupying the space of Si, B, and O atoms and resulting in longer distances among the locations of peaks. In summary, these findings verify the optimal choice of 1 wt.% carbon for producing foam glass with smaller pores and a uniform pore structure, thereby, leading to superior compressive strength.

Because 1 wt.% carbon was selected, we proceeded to investigate different allotropes of carbon (graphite and carbon black) as foaming agents to attain preferable foaming effects. The graphite-agent foam glass sample shows a higher density and strength but with a lower porosity (Table 2), which might be due to the higher oxidation temperature of graphite. Furthermore, the prepared foam glass samples with graphite as the foaming agent are difficult to cut and process, which is another disadvantage as compared with using carbon black.

### 2.2. Chemical and Thermal Stability of the Foam Glass Samples with Carbon as Foaming Agents

To investigate the corrosion-resisting ability of the foam glass samples with carbon black as a foaming agent for water-based applications, we measured the weight changes of cubic (side length 10 mm) samples immersed in aqueous solution with different pH values (Table 3). This finding shows that the pH and immersion time can affect the weight change significantly. The acid solution leads to decreased weight of the sample; the stronger the acid is, the greater the sample weight loss. In contrast, water leads to increased weight of the samples. In addition, the sample weights remain stable after a sufficient time of immersion (60 days), indicating a stable state with corrosion resistance. More importantly, the changes in the sample weights remain within 0.7%, which poses limited negative effects to the usability of the foam glass samples, showing strong corrosion resistance and chemical stability. In acid solution, the hydrogen ion is more active, which hydrolyzes the foam glass into salt and promotes weight loss. Nonetheless, the addition of Na_2_O and K_2_O could limit the motion of the alkali metal ions by the mixed alkali effects [22], thereby, strengthening the corrosion resistance and chemical stability of the glass network.

In addition, thermal stability is of special interest when foam glass is extensively used in places with high or fluctuating temperatures. The thermal stability of the samples was measured by calculating the thermal coefficient of linear expansion (Table 4). The averaged thermal coefficient of linear expansion from 27 °C to 100 °C is 11.10 × 10^−6^/°C. The deviation of the coefficient is less than 1 × 10^−6^/°C, which shows thermal stability when subjected to heat or temperature fluctuation in a relatively wide temperature range. In summary, the chemical and thermal stability of the foam glass samples show great promise for long-term use when subjected to corrosion and heat.

### 2.3. Foaming Effect of CaSO_4_ and Its Impact on the Physical and Thermal Properties of the Foam Glass Samples

**Figure 3:**
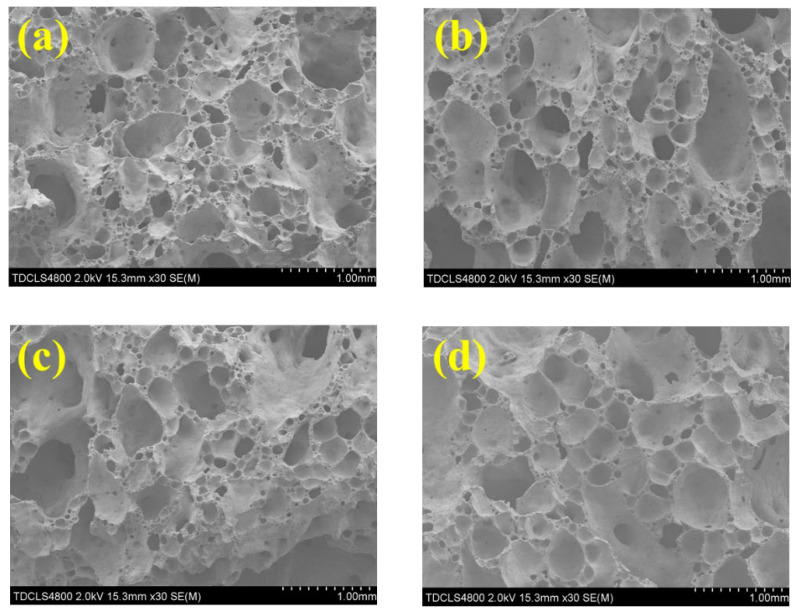
Pore surface morphology of the foam glass samples by SEM with: (**a**) 1 wt.%; (**b**) 2 wt.%; (**c**) 3 wt.%; (**d**) 4 wt.% CaSO_4_, under a sintering temperature of 775 °C.

CaSO_4_ is one type of foaming promoter that can significantly improve the foaming ability of foam glass materials [17]. We added different amounts (1/2/3/4 wt.%) of CaSO_4_ into the raw material mixtures to investigate the surface morphology (Figure 3) and thermal properties (Table 5) under a low sintering temperature of 775 °C The samples prepared with 4 wt.% CaSO_4_ (Figure 3d) show the most uniform, shape-ordered pore structure with an averaged pore diameter of ~300 μm. However, the samples prepared with 1/2/3 wt.% CaSO_4_ (Figure 3a–c) lead to an averaged pore diameter of ~200 μm with nonuniform pore structures. In general, the addition of CaSO_4_ reduces the pore diameter and leads to many small pores. In addition, the denser pore structure results in thinner and more brittle pore walls, which are expected to yield lower compression strength, corresponding well with existing works [17]. The reason might be that, although CaSO_4_ generates more gas in the glass melts, the formation of CaO increases the viscosity of the glass melts. This prevents liquid motion and, thus, leads to difficulties in pore merging and growing, thereby, yielding many small pores.

The thermal coefficients of linear expansion from 30 °C to 300 °C were calculated to measure the thermal stability of the foam glass samples with 4 wt.% CaSO_4_. The coefficients remain stable among different temperature ranges with an average value of 10.91 × 10^−6^/°C from 30 °C to 300 °C and the lowest value of 8.78 × 10^−6^/°C from 100 °C to 150 °C. The coefficient increases a little when it is above 150 °C. These results show the thermal stability of the prepared foam glass samples when subject to heat or temperature fluctuations in a relatively wide temperature range.

### 2.4. Foaming Effect of Na_2_HPO_4_ and Its Impact on the Physical and Mechanical Properties of the Foam Glass Samples

Foam stabilizers such as Na_2_HPO_4_ [10,23] can reduce the pore nonuniformity of foam glass material, by which the mechanical strength of the product is significantly enhanced. Na_2_HPO_4_ under heat decomposes into P_2_O_5_, the P^5+^ of which forms [PO_4_] tetrahedra, constituting a continuous network structure with the [SiO_4_] tetrahedra. This increases the viscosity of the melting glass at high temperatures and amends the glass network, thereby, strengthening the mechanical integrity and stability.

**Figure 4:**
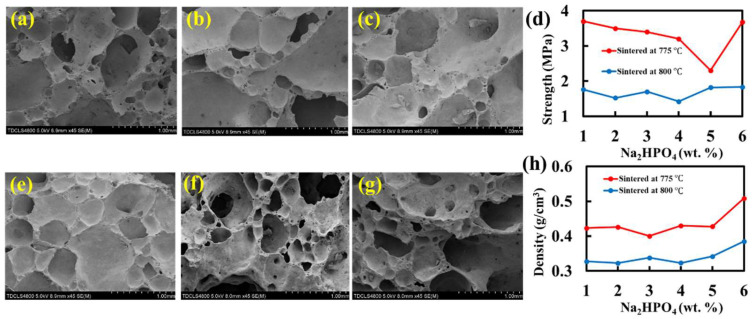
Pore surface morphology of the foam glass samples by SEM stabilized by: (**a**) 1 wt.%; (**b**) 2 wt.%; (**c**) 3 wt.%; (**e**) 4 wt.%; (**f**) 5 wt.%; (**g**) 6 wt.% Na_2_HPO_4_, under a sintering temperature of 775 °C. The trend of (**d**) compressive strength and (**h**) density as a function of different amounts of Na_2_HPO_4_ under two sintering temperatures of 775 °C and 800 °C.

The pores are gradually stabilized as the amount of stabilizer increases from 1 wt.% to 4 wt.% (Figure 4 a–c,e) at a sintering temperature of 775 °C. The pore diameter in the pore walls decreases with the addition of Na_2_HPO_4_. The opened and large pores on the pore walls gradually disappear, the diameters of the pores become smaller, and the pores distribute more uniformly. When the amount of stabilizer is 4 wt.% (Figure 4e), the pores on the pore walls almost disappear. Under this condition, the pores are mostly closed and uniform with smooth pore walls. The average pore diameter is ~0.5 mm. When the amount is 5 wt.% or 6 wt.% (Figure 4f,g), some small pores appear again, which is not beneficial to the properties of foam glass. This should be due to that when the amount of Na_2_HPO_4_ is overly large, [PO_4_] amends the glass network and increases the viscosity of the glass melts, preventing the liquid motion and gas pore growing. An overly large amount of Na_2_HPO_4_ could also lead to large product density which is another disadvantage.

The compressive strength and density of the foam glass samples (Figure 4d,h) with Na_2_HPO_4_ were measured under two selected sintering temperatures, i.e., 800 °C and 775 °C. For 800 °C, the compressive strength with Na_2_HPO_4_ (at maximum 1.88 MPa) is lower than without it (3.22 MPa) [17]. This implies that despite the stabilization of the pore structures with Na_2_HPO_4_, the pore walls become more brittle due to overly small pores. It should be noted that, although usually smaller and more uniform pores could lead to stronger foam glass materials, overly small pores may yield more brittle pore walls, thereby, causing lower strength. Nonetheless, at 775 °C, the strength is remarkably improved to 3.67 MPa at maximum and 2.38 MPa at minimum, which shows that a relatively lower sintering temperature of 775 °C may lead to improved mechanical performance. This corresponds well with previous work [15]. Next, the densities for 800 °C are found to vary in the range of 0.3–0.4 g/cm^3^; while for 775 °C, they vary in the range of 0.4–0.5 g/cm^3^. Therefore, when the sintering temperature is decreased from 800 °C to 775 °C, the strength increases by 95.21%, while the density only increases 23.67%. This indicates a preferable sintering temperature of 775 °C for preparing stronger foam glass materials as compared with 800 °C.

## 3. Discussion

In summary, we investigated the foaming effect of several foaming agents, promoter, and stabilizer and its corresponding impact on the physical and mechanical properties of borosilicate foam glass samples. The foaming effect was further elucidated by characterizing the structural configuration at the molecular level with MD simulations. More specifically, to possibly enhance the foaming effect, foaming agents of dextrin and a mixture of dextrin and carbon, and different carbon allotropes were investigated by characterizing surface morphology with SEM and physical and mechanical property, in which the 1 wt.% carbon black was found to be the optimal choice for a balanced material property. RDFs and NDPs were then computed to attain a molecular-level understanding of the foaming effect, where smaller pores and more uniform pore structure were found to benefit the mechanical behaviors of foam glass samples. Next, the foam glass samples showed excellent chemical and thermal stability with carbon as the foaming agent. Finally, the foaming effect of the foaming promoter CaSO_4_ and the foaming stabilizer Na_2_HPO_4_ were explored, where they were both found to effectively enable a more uniform pore structure.

Improving foaming effect via rationally selecting foaming agents, promoters, and stabilizers can enable more efficient production of foam glass materials with higher mechanical performance and lower cost. More importantly, the exploration of different foaming facilitators can produce foam glass materials with diversified structural characteristics, which can meet diversified engineering needs. Although pure chemicals were used in this work, these findings may guide or be referred to for future related foam glass manufacturing using recycling glass wastes. For instance, with X-ray fluorescence analyses [8,24,25], we can roughly determine whether a glass waste mixture has a stochiometric ratio close to the preferred ratio of pure chemical raw mixtures. Furthermore, systematic approaches for preparing low-cost and high-performance foam glass materials remain scarce, owing to the lack of a fundamental understanding of the structure–property–process relationships at the molecular level, despite decades of efforts. MD simulations may be an effective approach to understanding key molecular-level phenomena. These theoretical understandings and the resulting computational design could lead to significant achievement in efficient experimental and industrial production of high-performance foam glass materials, and therefore, avoids the conventional trial-and-error approaches.

## 4. Materials and Methods

### 4.1. Experimental Methods

All the experimental raw materials and reagents are industrially pure and commercially available. Basic raw chemicals used in this study included H_3_BO_3_ (Tianjin Yishang Group Co., Ltd., Tianjin, China), SiO_2_ (quartz-phase, Dahan Minerals (Xinyi) Co., Ltd., Xuzhou, China), Na_2_CO_3_, K_2_CO_3_, and Al_2_O_3_ (Hehai Science Technology & Engineering Co., Ltd.). These raw chemicals were well blended as a mixture with a basic stochiometric ratio of H_3_BO_3_ 13 wt.%, SiO_2_ 60 wt.%, Na_2_CO_3_ 17 wt.%, K_2_CO_3_ 5 wt.%, Al_2_O_3_ 5 wt.%. This basic composition was fixed throughout the entire study and experimental variables were given as follows: foaming agents included carbon black (Tianjin Chemical Reagents Co., Tianjin, China), graphite (Beijing Hanjie Science & Technology Development Co., Ltd., Beijing, China), dextrin (Tianjin Chemical Reagents Co.), and other addition materials of CaSO_4_ and Na_2_HPO_4_ (Tianjin University Kewei, Tianjin, China). The reagent was sulfuric acid (Tianjin Chemical Reagents Co., pH = 1 and pH = 3). The experimental facilities included electronic scales (PL203, METTLER TOLEDO Group), an electrically heated thermostatic drying oven (DH-204, Tianjin Middle Ring Experiment Electric Cooker Co., Ltd., Tianjin, China), a ball mill (DH48S, Xinling Electrical Co., Ltd., Wenzhou, China), a cased resistance-heated furnace (SSX-8–16, Shanghai Shiyan Electric Furnace Co., Ltd., Shanghai, China), a thermal expansion tester (Germany NETZSCH DIL-402C, Selb, Germany), quartz mortar, saw, abrasive papers, 250-mesh sieve, vernier caliper, graphite crucible, spoon, and brush. The experimental procedures, in order, involved: raw materials scaling, blending, wet grinding, drying, dry grinding, sieving, molding, heating, sintering, annealing, and characterizing. The detailed heat treatment consisted of the following: After the molding of the mixtures in the graphite crucible, the mixtures were preheated at 400 °C at a rate of 3 °C/min in a furnace for 30 min to allow for sufficient heating. Then, the mixture was heated to 775/780 °C at a rate of 4 °C/min, and preserved at this temperature for 30 min. Next, the mixture was annealed to 600 ℃ at a rate of 3 °C/min, and preserved at this temperature for 30 min. Finally, the mixture was naturally cooled for at least 24 h to room temperature, before the characterization of the products. Following the abovementioned procedures, the obtained products were confirmed to be glass by XRD patterns [17]. Thermogravimetric and differential scanning calorimetry (TG-DSC, STA449C, Netzsch Gerätebau, Bavaria, Germany) were used to measure the thermal behaviors of the foam glass samples. Scanning electron microscopy (SEM, S-4800, Hitachi, Tokyo, Japan) and optical microscopy were used to characterize the surface morphology of the foam glass samples.

After removing the foam glass samples from the furnace, a saw and abrasive papers were used to cut and polish the samples into cubic shapes with a side length of 1 cm. Then, we used a vernier caliper to measure the length, width, and height of the cubic, 10 times, to obtain the average volume *V*. The dried samples were put onto an electronic scales to obtain a weight $M_{0}$. Next, the samples were soaked in deionized water for 2 h at room temperature. After 2 h, the wet sample surfaces were dried with water-absorbing papers and put on the electronic scales again to obtain a weight $M_{1}$. The measurement of weight change in the sulfuric acid and deionized water for testing chemical stability was obtained similarly to these procedures, except that the soaking time was much longer. Finally, the volume-absorption rate can be calculated as follows:

(1) $$W_{V}=\frac{\Delta V}{V}=\frac{M_{1}-M_{0}}{V}$$

where $M_{0}$ and $M_{1}$ are weights before and after soaking, respectively, in grams; ΔV is the volume of the absorbed water of the fully soaked samples, in cm^3^; and *V* is the average volume, in cm^3^. Considering that water has a density of 1 g/cm^3^, the water volume change is equal to its weight change in magnitude (Equation (1)). Bulk density is calculated to be the weight in a unit volume:

(2) $$\rho_{\nu }=\frac{M_{0}}{V}$$

where $\rho_{\nu }$ is the apparent bulk density in g/cm^3^. The true density, $\rho_{t}$, is defined as the weight of the fully ground and dried samples of a unit volume without pores inside. Porosity is an important metric for measuring the compactness of the samples and is categorized into opened, closed, and total porosity. Total porosity is defined as:

(3) $$P_{t}=1-\frac{\rho_{v}}{\rho_{t}}$$

where $\rho_{t}$ is the true density, in g/cm^3^.

A universal tester was used to perform the compressive strength test of the foam glass samples. The stressed area *S* was readily available by virtue of the cubic shape of the samples. A low strain rate of 1 mm/min with the universal tester was used and a maximum compressive force *P* was obtained before fracture. Therefore, the compressive strength is calculated as:

(4) $$S_{c}=\frac{P}{S}$$

where *P* is the maximum compressive force, in a unit of N; *S* is the stressed area, in a unit of mm^2^; and $S_{c}$ is the compressive strength, in a unit of MPa. All the test results were averaged by repeatedly testing 10 samples under the same condition. A thermal expansion tester was used with a temperature heating rate of 5 °C/min. The thermal coefficient of linear expansion is calculated as:

(5) $$\alpha_{l}=\frac{dL}{LdT}$$

where *L* is the length of the sample at a temperature *T,* in a unit of mm; *dL* is the length change corresponding to a temperature change of *dT*; and $\alpha_{l}$ is in a unit of 10^−6^/°C.

### 4.2. Computational Methods

AAMD simulations have been successfully used to characterize the physical and mechanical properties of many glass and ceramic materials [26,27]. Extensively developed potential forcefields [27,28,29] were used to compute the structural configuration of the foam glass at the molecular level. Details of the forcefield and our validation of using it in our study can be found in previous work [17]. The interatomic potential is described with two-body Buckingham potential as:

(6) $$U_{ij}\left(r_{ij}\right)=\frac{z_{i}z_{j}}{r_{ij}}+A_{ij}exp\left(\frac{-r_{ij}}{\rho_{ij}}\right)-\frac{C_{ij}}{r_{ij}^{6}}$$

where $r_{ij}$ represents the distance between the *i*-th and *j*-th atoms; $z_{i}$ represents the effective partial charge of the *i*-th atom; and $A_{ij}$, $\rho_{ij}$, and $C_{ij}$ are the fixed energy parameters for pairwise interactions, which correspond to electrostatic interactions, short-range electronic repulsion, and van der Waals interactions, respectively. The interatomic potential function parameters and fixed elemental partial charge are given in Table 6; Table 7, respectively. The carbon dioxide gas is described with a summation of short-range interaction between carbon and oxygen atoms and electrostatic interactions:

(7) $$U_{ij}\left(r_{ij}\right)=\underset{i}{\sum }\underset{j}{\sum }u\left(r_{ij}\right)+\underset{i}{\sum }\underset{j}{\sum }\frac{z_{i}z_{j}}{r_{ij}}$$

where the short-ranged interactional term *u* is described with Lennard–Jones (LJ) potential. The LJ potential is:

(8) $$u\left(r_{ij}\right)=4\varepsilon_{ij}\left[{\left(\frac{\sigma_{ij}}{r_{ij}}\right)}^{12}-{\left(\frac{\sigma_{ij}}{r_{ij}}\right)}^{6}\right]$$

where $\varepsilon_{C-C}=0.057\;Kcal/mol\;\mathrm{and}\;\varepsilon_{O-O}=0.164\;Kcal/mol$ are the dispersion energy, and $\sigma_{C-C}=2.7918\;Å$, and $\sigma_{O-O}=3.0\;Å$ are the equilibrium distance at which the LJ potential energy is zero between the two interacting atoms. The bond length between the carbon and oxygen atoms in a carbon dioxide molecule is 1.163 Å. In addition, we set a cutoff of 11 Å for the electrostatic and short-range interactions. We used the particle-particle particle-mesh algorithm [30] with an accuracy of 10^−5^ for the long-range electrostatic interactions.

For the simulation system, we constructed the model on the basis of the experimental raw material mass compositions (basic stochiometric ratio): H_3_BO_3_ 13 wt.%, SiO_2_ 60 wt.%, Na_2_CO_3_ 17 wt.%, K_2_CO_3_ 5 wt.%, and Al_2_O_3_ 5 wt.%. We modeled a mixture system with a mass of $4.98\times 10^{-19}$ g. Accordingly, with the molecular mass of these chemical constituents, we obtained the molar fraction of all the chemical constituents of H_3_BO_3_ 13.65%, SiO_2_ 65.02%, Na_2_CO_3_ 10.40%, K_2_CO_3_ 2.34%, Al_2_O_3_ 3.19%, and CO_2_ 5.40%. Therefore, the totals of the constituent atoms were: H 267, B 89, Si 989, Na 158, K 36, C 179, Al 60, and O 2790, with a total of 4568 atoms. We had two basic assumptions to realistically model the experimental condition: (1) the carbon was assumed to be totally oxidized into carbon dioxide; (2) the raw mixtures were mixed sufficiently and reacted adequately. To achieve a glass density of 0.346 g/cm^3^ (Table 2), a simulation box dimension of $\sqrt[{3}]{\frac{4.98\times 10^{-19}\;g}{0.346\;g/cm^{3}}}=11.29$ nm was required.

We used the Large-scale Atomic/Molecular Massively Parallel Simulator (LAMMPS) [31,32] to carry out the AAMD simulations. The benchmarking system with 1 wt.% carbon added was modeled with a cubic simulation box (dimension length 11.29 nm) consisting of 4568 atoms with a randomly generated initial structure satisfying the required density of 0.346 g/cm^3^ (Table 2). The system was melted at 780 °C, first, in a canonical ensemble (*NVT*) for 50 ps, followed by an isothermal-isobaric ensemble (*NPT*) for another 100 ps. This ensured that the system was not affected by the initial configurations to mimic an experimental condition. Then, the system was slowly cooled down to room temperature, and then equilibrated for a sufficient length of time (100 ps). The last 20 simulation frames with an increment of 1 ps between each frame were statistically averaged for further analysis. The choice of these parameters had been validated in our previous work [17]. A fixed timestep of 1 fs was used. Periodic boundary conditions at all dimensions were used throughout all the simulations. Radial distribution function (RDF) and number density profiles (NDP), attained from the abovementioned statistically averaged molecular configurations, were computed to describe the molecular-level structural feature of the foam glass samples. NDP is defined as the number distribution of a certain type of atoms/particles that appear along a certain specified crystallographic direction. It is used to characterize the distribution of a certain type of atoms/particles along a random direction [18]. In this study, 50 × 50 × 50 bins were used to divide the simulation box and determine if the certain type of atoms/particles appeared within a small bin along the specified direction, finally leading to an atom/particle density distribution along the specified direction.

## Figures and Tables

**Table 1:** Comparison of the oxidation-type foaming agent (dextrin) and a mixture of dextrin and the reduction-type foaming agent (carbon) [17].

Foaming Agents (wt.%)	Strength (MPa)	Density (g/cm^3^)	Porosity	Volume-Absorption Rate
Dextrin (2.5)	2.254	0.306	85.42%	1.15%
Dextrin (0.5) and carbon black (1.75)	2.854	0.339	82.52%	2.15%

**Table 2:** Comparison of two allotropes of carbon as foaming agents.

Foaming Agents	Strength (MPa)	Density (g/cm^3^)	Porosity
Graphite (1 wt.%)	5.12	0.536	74.47%
Carbon Black (1 wt.%)	3.24	0.346	83.52%

**Table 3:** Chemical stability (weight of the foam glass samples) after days of immersion in sulfuric acid (pH = 1 and pH = 3) and deionized water (pH = 7). “+“ Denotes weight increase and “−“ denotes weight decrease.

pH of the Solution	15 Days	30 Days	45 Days	60 Days
1	−0.30%	−0.50%	−0.60%	−0.60%
3	−0.30%	−0.50%	−0.57%	−0.60%
7	+0.45%	+0.55%	+0.70%	+0.70%

**Table 4:** Thermal coefficient of linear expansion in different temperature ranges.

Temperature Ranges (Celsius)	Averaged Thermal Coefficient (10^−6^/°C)
27–50	10.63
50–75	11.55
75–100	11.03

**Table 5:** Thermal coefficient of linear expansion in different temperature ranges with 4 wt.% CaSO_4_.

Temperature Ranges (Celsius)	Averaged Thermal Coefficient (10^−6^/°C)
30–100	9.56
100–150	8.78
150–200	12.21
200–300	12.21
30–300	10.91

**Table 6:** Potential function parameters used in this study [27,28].

Bond	$A_{ij}\;\left(eV\right)$	$\rho_{ij}\;\left(Å\right)$	$C_{ij}\;\left(eV\cdot {Å}^{6}\right)$
B–O	206,941.81	0.124	35.0018
Si–O	50,306.10	0.161	46.2978
O–O	9022.79	0.265	85.0921
B–B	484.40	0.35	0
B–Si	337.70	0.29	0
Na–O	120,303.80	0.17	0
Ca–O	155,667.70	0.178	42.2597
K–O	2284.77	0.29	0
Al–O	28,538.42	0.172	34.5778

**Table 7:** Fixed elemental partial charge [27,28].

Element	Partial Charge (*e*)
B	1.4175
O	−0.945
Si	1.89
Na	0.4725
K	0.4725
Al	1.4175
Ca	0.945
C	0.5888

## Data Availability

Data available on request due to restrictions. The data presented in this study are available on request from the corresponding author.
